# OD34 Consideration Of Medical Device Characteristics In Health Technology Assessment: A Gap In The Methods Guidelines And In Practice

**DOI:** 10.1017/S026646232400165X

**Published:** 2025-01-07

**Authors:** Rituparna Basu, Simon Eggington, Natalie Hallas, Liesl Strachan

## Abstract

**Introduction:**

It is well accepted that medical devices (MDs) and procedures have several unique characteristics compared to pharmaceuticals, such as learning curve (LC), incremental innovation (II), dynamic pricing (DP), and organizational impact (OI). The objective of this study was to determine the extent to which these MD characteristics are routinely assessed by health technology assessment (HTA) agencies and incorporated in their guidelines and reports.

**Methods:**

Three approaches were taken. First, a review of the most recent HTA methods guidelines from 13 selected HTA agencies and five HTA networks was undertaken. Next, HTA reports from these agencies were reviewed for inclusion of MD-specific characteristics for 16 selected MDs, and finally, a narrative literature review on this topic was conducted.

**Results:**

Twelve of thirteen included HTA organizations and some HTA networks (2/5) have either published general or MD-specific method guidelines, while several addressed MD-specific characteristics. The National Institute for Health and Care Excellence (NICE) included all four MD characteristics in their guidelines, but this did not equate to their inclusion in published HTA evaluations. EuNetHTA described the inclusion of LC (within patient safety) and OI within their guidance. The results highlight a lack of consistency among HTA organizations. For the narrative review, 10/149 articles were reviewed. Most provided recommendations on challenges faced by HTAs, proposed steps to address uncertainties around MD characteristics and reported a lack of methodological guidance for evaluating MDs.

**Conclusions:**

A lack of inclusion of MD characteristics in HTA is a complex interplay of several important factors. For these characteristics to become a formal part of HTA of MDs in the future, clear guidance and frameworks are required to enable manufacturers to develop appropriate evidence and for HTA practitioners to assess their impact more broadly.

